# Endocrine-Disrupting Chemicals and Infectious Diseases: From Endocrine Disruption to Immunosuppression

**DOI:** 10.3390/ijms22083939

**Published:** 2021-04-11

**Authors:** Elikanah Olusayo Adegoke, Md Saidur Rahman, Yoo-Jin Park, Young Ju Kim, Myung-Geol Pang

**Affiliations:** 1 Department of Animal Science & Technology and BET Research Institute, Chung-Ang University, Anseong 17546, Korea; adegokeelkanah@gmail.com (E.O.A.); shohagvet@gmail.com (M.S.R.); legend0508@naver.com (Y.-J.P.); 2 Department of Obstetrics and Gynecology, College of Medicine, Ewha Womans University, Seoul 07985, Korea; kkyj@ewha.ac.kr

**Keywords:** endocrine disrupting chemicals, COVID-19, comorbid diseases, immune dysfunction

## Abstract

Endocrine-disrupting chemicals (EDCs) are hormonally active compounds in the environment that interfere with the body’s endocrine system and consequently produce adverse health effects. Despite persistent public health concerns, EDCs remain important components of common consumer products, thus representing ubiquitous contaminants to humans. While scientific evidence confirmed their contribution to the severity of Influenza A virus (H1N1) in the animal model, their roles in susceptibility and clinical outcome of the coronavirus disease (COVID-19) cannot be underestimated. Since its emergence in late 2019, clinical reports on COVID-19 have confirmed that severe disease and death occur in persons aged ≥65 years and those with underlying comorbidities. Major comorbidities of COVID-19 include diabetes, obesity, cardiovascular disease, hypertension, cancer, and kidney and liver diseases. Meanwhile, long-term exposure to EDCs contributes significantly to the onset and progression of these comorbid diseases. Besides, EDCs play vital roles in the disruption of the body’s immune system. Here, we review the recent literature on the roles of EDCs in comorbidities contributing to COVID-19 mortality, impacts of EDCs on the immune system, and recent articles linking EDCs to COVID-19 risks. We also recommend methodologies that could be adopted to comprehensively study the role of EDCs in COVID-19 risk.

## 1. Introduction

Endocrine-disrupting chemicals (EDCs) are ubiquitous in common consumer products, processed food, drinking water, food packaging, and plastic materials, and humans are regularly exposed to EDCs via oral, inhalation, transdermal, and parenteral routes [1,2]. The most common EDCs include bisphenols, phthalates, arsenic, pesticides, dioxins, and perfluorinated compounds. Due to their ability to interfere with hormone-driven processes and their toxicity, EDCs have become a major research focus in the last few decades. Research findings so far have confirmed EDCs as cogent contributors to the risk of the underlying comorbidities of chronic and infectious diseases [1,2,3,4,5,6,7]. EDCs such as arsenic and 2,3,7,8-tetrachlorodibenzo-p-dioxin (TCDD) were reported to impair the host immune response to H1N1, leading to enhanced pulmonary inflammation and increased mortality [3,4]. Meanwhile, both COVID-19 and influenza have some similarities being contagious respiratory diseases [8].

Since its outbreak in late December 2019 in Wuhan city, China, the COVID-19 pandemic has spread to all continents [9]. As of 23 February 2021, there were 112,258,917 confirmed cases, 2,485,295 deaths, and 221 affected countries [10]. Early symptoms displayed by patients with COVID-19 include fever, cough, fatigue, and headache [11]. Older people and those with underlying comorbidities are prone to critical illness or death [12]. Among the first 41 patients with laboratory-confirmed COVID-19 as of 2 January 2020 at Jinyintan hospital, Wuhan, 32% had underlying comorbidities, i.e., diabetes mellitus, hypertension, and cardiovascular diseases [11]. According to another report, 269 (49.1%) of 548 patients with COVID-19 admitted to Tongji Hospital, China, from 26 January to 5 February 2020 were severely ill. Among these patients, 166 (30.3% of the total) and 83 (15.1% of total) had hypertension and diabetes as underlying comorbidities, respectively [5]. Similarly, 48% of 191 COVID-19 patients from Jinyintan and Wuhan Pulmonary Hospitals, China, were reported to have underlying comorbidities, with 30% having hypertension and 19% having diabetes [13]. Among 663 patients at Wuhan University Hospital from 11 January to 6 February 2020, a higher percentage (67.4%) of underlying comorbidities was the explanation for lack of improvement, severity, and mortality in COVID-19 disease [14]. Diabetes, high blood pressure, obesity, hypertension, low immunity, cardiovascular disease, and kidney and liver diseases as comorbidities result in extremely severe COVID-19 [12,13,15,16]. Meanwhile, long-term exposure to EDCs might be responsible for the development and progression of these diseases. Although there are variations in the percentage of patients with underlying medical conditions in different regions worldwide, metabolic, circulatory, and endocrine diseases are the most common comorbidities.

Recently, it was reported that environmental chemical related disorders have common pathogenic mechanisms with infectious diseases such as coronavirus-related diseases [17]. In a separate report, it was revealed that exposure to toxic substances played vital roles in the COVID-19 pandemic [18,19]. Similarly, a computational systems biology approach identified Th17 and AGE/RAGE signaling pathways as links between EDCs and COVID-19 severity [20].

In this article, we review the recent literature on the roles of EDCs in comorbidities contributing to COVID-19 mortality, impacts of EDCs on the immune system, and recent articles linking EDCs to COVID-19 risks. We also recommend methodologies that could be adopted to comprehensively study the role of EDCs in COVID-19 risk.

## 2. Sources of EDCs Exposure

**Figure 1:**
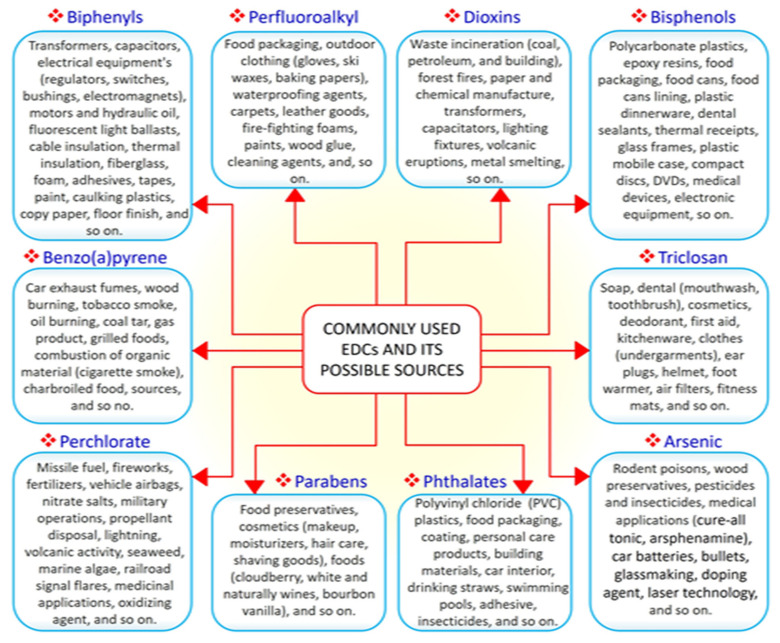
The 10 commonly used endocrine-disrupting chemicals (EDCs) and their common sources.

EDCs are natural or synthetic compounds that can act as hormones by manipulating and compromising several mechanisms of the endocrine system to produce serious effect on the overall health of both humans and animals [21,22,23]. EDCs such as bisphenols (e.g., bisphenol A, BPA) and phthalates are essential chemicals used as raw materials in plastic industries [24]. Other groups of EDCs include dioxins, perchlorate, perfluoroalkyl, and polyfluoroalkyl substances, phytoestrogens, polybrominated diphenyl ethers (PBDE), polychlorinated biphenyls (PCB), and triclosan, which constitute components of many consumer and household products, including foods, resulting in widespread human exposure [25,26]. A search of the literature demonstrated that the vast majority of EDCs are widely and increasingly being used worldwide. Derivatives of BPA (bisphenol S, bisphenol F, and bisphenol E) are used in the production of polycarbonate plastics, epoxy resins, food packaging, dental sealants, and thermal receipts [27,28,29]. High-molecular-weight phthalates such as di (2-ethylhexyl) phthalate (DEHP), di-isononyl phthalate di-isodecyl phthalate, and benzylbutyl phthalate are used in making polyvinyl chloride (PVC) plastics, medical devices, pharmaceutical coatings, food packaging, car interiors, drinking straws, and adhesives [30]. Low-molecular-weight phthalates such as diethyl phthalate, di-n-butyl phthalate, and di-iso-butyl phthalate are used in making perfumes, deodorants, nail polish, and insecticides [30]. PBDE, perchlorate, triclosan, and polychlorinated biphenyls are used in making furniture foam, fireworks, liquid body wash, and hydraulic fluids, respectively [31]. As the most common EDCs, BPA and phthalates can be ingested along with food and beverages packaged in containers containing them. The ubiquitous distribution of EDCs makes it possible for humans to be exposed to EDCs via the transdermal, oral, inhalation, and parenteral routes [32,33]. A summary of commonly used EDCs and their exposure sources is compiled in Figure 1.

## 3. EDC-Related Diseases

**Figure 2:**
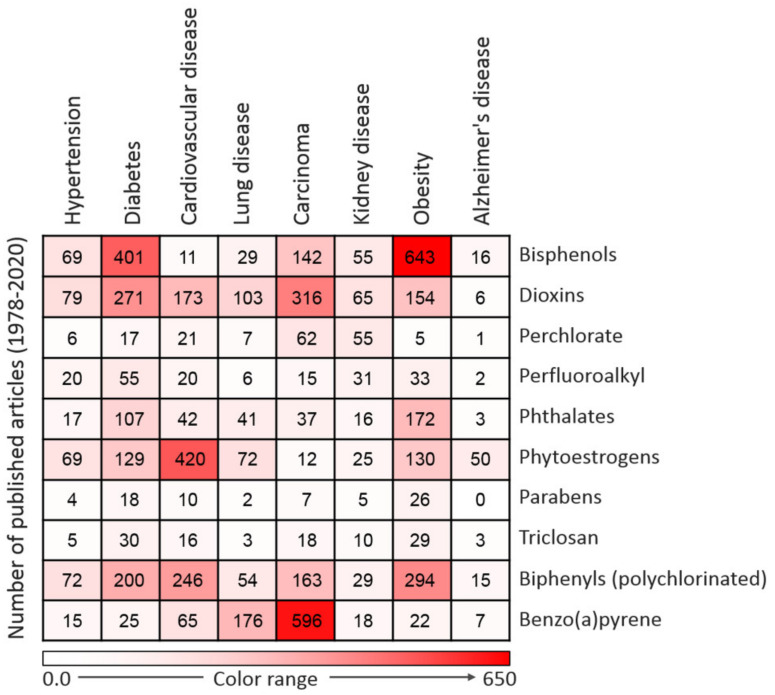
Heatmap representing the number of articles published on the 10 commonly used endocrine-disrupting chemicals (EDCs) in association with particular diseases. Data were collected by a thorough search of the Web of Science database. Only peer-reviewed papers published in English from 1978 to 2020 on specific EDCs and diseases were included.

EDCs have notable effects on biological systems. Exposure to EDCs alters the endocrine system of the body and causes serious metabolic, neurological, cardiovascular, and immunological effects in humans and animals, including wildlife [34]. Generally, EDCs via binding with estrogen receptors can activate transcription factors such as protein-1 (AP-1), nuclear factor-kappa B (NF-κB), and specificity factor-1 (Sp1), which are involved in the pathogenesis of inflammation which accounts for the onset of many chronic and comorbid diseases [34,35,36]. The synthesis of natural hormones may be obstructed and inhibited by EDCs [34]. The effect of exposure to EDCs during the critical stage of development could be latent until adulthood [35]. It has been proven that EDCs not only affect the exposed person but also their offspring and successive generations [36]. Research has established that exposure to EDCs is positively correlated with the escalated emergence of cancer, obesity, Parkinson’s disease, and other diseases [35,37,38]. These effects compelled the Endocrine Society to release a publication in 2009 showing EDCs as important public health concerns [39]. A summary of major EDC-related diseases is presented in Figure 2.

### 3.1. Metabolic Disorders and Obesity

Recently, a number of studies reported an association between EDC exposure and the stimulation of adipogenesis and weight gain [40]. Obesogens EDCs can promote weight gain through the proliferation and subsequent storage of fat cells [41].

Obesogens such as BPA, PCBs, dioxins, and phthalates can also promote weight gain by compromising energy homeostasis and the basal metabolic rate [40,42]. At a molecular level, obesogens EDCs act by interfering with nuclear transcriptional regulators that control lipid flux and/or adipocyte proliferation/differentiation, especially the peroxisome proliferator-activated receptors (PPARα, PPAR-δ, and PPAR-γ) and steroid hormone receptors. PPARs act by heterodimerization with retinoid X receptors (RXRs), and the activation of RXR-PPARγ potentiates the differentiation of adipocyte progenitors and preadipocytes in adipose tissue. Consequently, fat/lipid biosynthesis and storage are promoted and finally result in obesity [40,42]. Obesity-related health conditions are prevalent in many industrialized countries where EDCs are produced and used in large quantities [43]. In 2013, 90 million (28.6%) cases of obesity were found in the US population of 315 million, and 78 million of these cases were found in adults [43]. It has been extrapolated that approximately 40% of the world’s population will have obesity by 2030 [44]. Obesity is known as a predisposing factor for several health conditions, including diabetes and hypertension [44]. An important cause of insulin resistance (IR) is oxidative stress, and elevated oxidative stress is induced by obesity via the excessive generation of mitochondrial energy [45]. Further, IR results in elevated circulating glucose levels, which in turn worsens the generation of oxidative stress [46], and type 2 diabetes and vascular disease may result from this vicious cycle [44]. A recent study also found a positive correlation between Alzheimer’s disease and IR [47].

### 3.2. Diabetes

To address the public concern that exposure to EDCs could contribute to widespread diabetes, researchers in the field of EDC research have conducted studies to investigate the association between EDC exposures and diabetes. Currently, there is confirmed evidence that diabetes is related to exposure to EDCs such as bisphenols, pesticides, and dioxins [48,49,50]. The same EDCs have been indicted in the development and progression of diabetes and obesity [40,42,51]. In a study conducted by Lee et al. [52] to investigate the association between persistent organic pollutants (a group of EDCs) and the prevalence of diabetes among 2016 adult participants, the serum concentration of persistent organic pollutants was found to be positively correlated with diabetes prevalence. In addition, exposure to many other groups of EDCs, such as polychlorinated biphenyls, bisphenols, dioxins, phthalates, and organochlorinated pesticides, has been reported to be associated with the risk of diabetes [53]. Persistent organic pollutants, BPA, dichlorodiphenyltrichloroethane (DDT), and phthalates have all been documented to play a diabetogenic role [54,55,56]. Association between EDC exposures and diabetes has also been demonstrated in experimental studies. Reduced insulin levels were observed in adult mice exposed to DDT during the perinatal window of development [57]. A similar study in which mice were exposed to BPA in utero resulted in the impairment of insulin secretion and glucose tolerance [58]. The molecular mechanism of EDCs action involves binding to the estrogen receptors α and β and thereby acting like estrogen. Long-term exposure to xenoestrogen EDCs hyperactivates the β-cells, leading to hyperinsulinemia. Consequently, there is a development of a condition known as insulin resistance/glucose intolerance, which is a significant cause of diabetes [48].

### 3.3. Hypertension and Cardiovascular Diseases

Hypertension (high blood pressure) is prevalent worldwide, mostly in older adults but also in individuals of different ages, and is considered to be among the leading causes of death in developed countries [59]. Hypertension related to hormone imbalance is common among humans. Vasodilation can be induced by estrogen via both genomic and non-genomic pathways. Endocrine disruptors are estrogenic and have been identified as risk factors contributing to the onset of hypertension [59]. The urinary concentration of BPA was shown to be positively correlated with hypertension in a survey conducted on 2588 individuals by the Thai National Health Examination Survey 2009 [60]. Another study conducted from 2008 to 2010 in Seoul, South Korea, reported a positive correlation between urinary BPA concentrations and blood pressure in 521 elderly citizens [61]. Urinary concentrations of BPA were reported to be correlated with increased diastolic blood pressure in 39 boys recruited from the Children Medical Center of Dayton, Ohio [62]. The evaluation of urinary BPA concentrations in 1380 participants of the 2003–2004 National Health and Nutritional Examination Survey (NHANES) also showed a positive correlation between elevated levels of urinary BPA and hypertension [63]. Consistently, cross-sectional studies have confirmed the effect of BPA on cardiovascular diseases. The analysis of NHANES data from 2003 to 2004 revealed a high correlation between the urinary concentration of BPA and cardiovascular diseases including myocardial infarction and coronary heart disease [64,65]. A urinary BPA concentration of 4.56 ng/mL significantly increased the risk of coronary artery disease [66]. Apart from the direct effect, hypertension is a prominent risk factor for cardiovascular diseases [67]. Since BPA affects blood pressure, it could cause cardiovascular disease via increased blood pressure. BPA can further affect the cardiovascular system because it is estrogenic; estrogen receptors are found in cells of the cardiovascular system, and estrogen is involved in vasodilation [65]. Estrogen is active both on vascular smooth muscle and endothelial cells where functional estrogen receptors have been found. By acting on vascular smooth muscle cells, EDCs activate K^+^ channels, leading to cell hyperpolarization, increase aortic stiffness, potentiate endothelial vasodilator function, and block the activation of Ca^2+^ channels, resulting in decreased intracellular Ca^2+^ concentration. Xenoestrogen EDCs promote vasodilation in humans by stimulating prostacyclin and nitric oxide synthesis and decreasing the production of vasoconstrictor agents [65]. Similarly, the onset of coronary heart disease, high blood pressure, and atherosclerosis in humans has been attributed to exposure to other EDCs [68].

### 3.4. Kidney Diseases

The nephrotoxic effect of most endocrine disruptors is a public health concern. Epidemiological studies have established a positive association between renal diseases and urinary BPA concentration in humans [69,70]. A cross-sectional study involving 3455 Chinese participants indicated that an average urinary BPA concentration of 0.81 ng/mL was correlated with an elevated risk of albuminuria [69]. Similarly, another cross-sectional study involving 710 children in the USA indicated that an average urinary BPA concentration of 0.91 mg/g is associated with albuminuria [70]. A renal function analysis of 184 children aged 10 years exposed to DEHP contaminated food revealed a significant association between exposure to DEHP and an elevated urine albumin/creatinine ratio [71]. The study further found that highly exposed children (with an average daily DEHP intake of 0.05 mg/kg/day) were 10.39% prone to the risk of microalbuminuria. Female mice exposed to 1500 and 6000 ppm of DEHP had a significantly higher proportion of chronic progressive nephropathy (CPN) cases than those in the control group [72]. In the same study, male and female mice exposed to the same concentration of DEHP experienced a reduction in kidney weight [72]. CPN in male rats was reported to be aggravated following exposure to 12,500 ppm of DEHP [72]. A separate report indicated that exposure to 3147 mg/kg/day of DEHP in mice resulted in the degeneration of the renal tubule and reduced kidney weight [73]. A rat model study, in which male Wistar rats were exposed to 50, 100, and 150 mg/kg of BPA for 5 weeks, found that BPA caused proteinuria, glomerular injuries, elevated serum, and urea creatinine in a dose-dependent manner [74]. EDCs, through steroidogenesis, accelerate kidney estrogen metabolism and stimulate the activity of cytochrome p-450 aromatase, resulting in oxidative stress. Some EDCs are competent to act directly on the kidney mitochondria, resulting in mitochondrial oxidative stress, dysfunction, and subsequently, whole organ damage [74]. Many EDCs such as BPA display nephrotoxicity and function as indicators for renal disease [75]. The current chronic kidney disease prevalence of 10–15% in the general population worldwide and the fact that EDCs contribute to this prevalence imply that EDCs constitute a threat to human wellbeing [76,77].

### 3.5. Cancer

Approximately 9.6 million deaths and 18.1 million new cases of various types of cancer were reported in 185 countries in 2018 [78]. The relationship between EDCs and cancer has been established for over a decade. Investigations have found several EDCs to be carcinogens that can promote the onset and progression of cancer through their hormone-like activities [79,80,81]. Recently, the development of cancer has been linked to microRNAs which are known to negatively regulate the expression of genes [82]. Estrogen-regulated onco-miR-21 has been shown to play a vital role in the development of breast cancer [83]. Dioxins, DEHP, and BPA can stimulate estrogen receptors, thus contributing to the development of estrogen-dependent cancers such as prostate and breast cancers [84]. EDCs can bind many nuclear receptors, such as estrogen receptors (ERα and β), GPR30, androgen receptor (AR), thyroid hormone receptors (TRα and β), estrogen-related receptor gamma (ERRγ) and glucocorticoid receptor (GR). The binding of EDCs to ER increases the proliferation and migration of several cancer cell types through a pathway involving Stat3 and ERK1/2 [79,80,81,82,83,84]. In a study conducted to evaluate the relationship between endocrine disruptors and the risk of thyroid cancer in 960 individuals (462 thyroid cancer patients and 498 control), increased risk of thyroid cancer was observed in persons who were exposed to EDCs compared to their control counterparts [85]. An investigation of the association between circulating serum EDC levels and mammographic breast density (an indicator of breast cancer risk) among 264 women from mammography clinics indicated that serum concentrations of BPA and mono-ethyl phthalate were positively correlated with mammographic breast density [86]. In another study, phytoestrogens, PCB, and dioxins were linked to the development of breast cancer, while arsenic and PCB were reported to significantly contribute to the incidence of prostate cancer [6,87].

### 3.6. Lung Diseases

The involvement of several EDCs in the development of human diseases via various routes and mechanisms has been established by several studies [23,88,89]. Exposure to EDCs can activate ERK1/2 via GPER/EGFR. Consequently, GPER/ERFR/ERK1/2 mediates the upregulation of matrix metalloproteinases (MMPs), collectively known as the gelatinases, which are generally crucial in inflammatory, infectious pathogenesis, neoplastic diseases, and the migration of lung cancer [35,88,89]. The insecticides used in many residential buildings, chemical components of the building, and furnishing materials are EDC sources of indoor exposure [90,91]. Low-level exposure to indoor EDCs is related to an increased risk of asthma. A cross-sectional study found that the risk of developing asthma was significantly increased in 815 pupils exposed to a mixture of EDCs (hexane, styrene, cyclohexanone, butylated hydroxytoluene, and 2-butoxyethanol) [92]. Cleaning agents and air fresheners containing EDCs have been identified as sources by which building occupants and cleaning personnel are exposed to a large number of airborne chemicals, consequently developing lung problems [93]. Exposure to approximately 3.4–17 mg/m^3^ of sodium tripolyphosphate and 14 mg/m^3^ of volatile organic compounds following carpet cleaning was reported to result in asthma and seizure in a 42-year-old woman [94]. In a separate occupational exposure case, all-female nurses of a hospital showed symptoms of asthma after handling a disinfectant solution containing EDCs [95]. A total of 2414 Finnish female cleaners developed asthma via exposure to cleaning agents, with a risk factor of 1.50 [96]. A similar finding was reported among Spanish cleaners; 28% of the cleaners developed asthma via exposure to EDC-containing kitchen cleaning agents and furniture polishing chemicals [97]. It has been demonstrated that a BPA concentration of 10^−4^ M promoted the proliferation and migration of A549 human lung cancer cells [98]. A risk of respiratory symptoms was linked to occupational exposure to PVC-containing fumes and residential exposure to PVC-contaminated dust [99]. Another human study indicated a positive correlation between asthma in children and the phthalate component of building dust [100]. In a study conducted on 56 children with asthma in Seoul, South Korea, urinary concentrations of phthalates (mono-[2-ethyl-5-hydroxyhexyl] phthalate and mono-[2-ethyl-5-oxohexyl] phthalate, metabolites of DEHP, and mono-n-butyl phthalate, a metabolite of di-n-butyl phthalate) were correlated with decreased pulmonary function, airway inflammation, and increased levels of fractional exhaled nitric oxide [101]. In addition, a clinical study aimed at investigating the association between the urinary concentration of EDCs and the function of the lung indicated that urinary concentrations of BPA and phthalates correlated with the impairment of lung function and oxidative stress in 411 persons aged > 58 years recruited for the study [102].

### 3.7. Neurodegenerative Diseases

Neurodegenerative diseases including Alzheimer’s disease affect people worldwide, especially older people. The number of Alzheimer’s disease cases is projected to increase to approximately 106 million in the next 30 years [103]. Although 70% of Alzheimer’s disease risk is attributed to genetics, environmental factors including EDC exposures account for the remaining 30% [104]. Insecticides, pesticides, dioxins, bisphenols, phthalates, and parabens are groups of endocrine disruptors that have been implicated in the development of Alzheimer’s disease. The nervous system of insect pests is the target of various pesticides; likewise, these chemicals are neurotoxic to humans [105]. Neurological diseases including Alzheimer’s disease have been attributed to exposures to pesticides [106]. A study that examined 7321 PCB-exposed workers found that serum PCB levels in exposed workers were approximately 10 times higher than those in community controls. Dementia, Parkinson’s disease, and neurological disease-related deaths were reported among highly exposed women [107]. An investigation into the link between pesticide exposure and Parkinson’s disease revealed a significant correlation: a significantly increased risk of Parkinson’s disease was observed in 13 of 23 cases, with a risk estimate of 2.4 [108]. An analysis of hospital records between 1998 and 2005 indicated that cases of Parkinson’s disease and Alzheimer’s disease were higher among people dwelling in an area of high pesticide use [109]. Following the investigation of a possible link between Alzheimer’s disease and dementia and exposure to pesticides among 5092 persons dwelling in Cache County, Utah, USA, a significantly high correlation was found between exposure to organophosphates and Alzheimer’s disease [110]. EDCs act on the pituitary gland or bind with estrogen/G-protein-coupled receptors involved in neurotransmission and, consequently, affect the central nervous system’s central dopamine neurons and monoaminergic neurons [79,80,81,82,83,84,88,89].

### 3.8. Immune Function

EDCs not only disrupt hormone activities but are also known to alter the function of the immune system. Human epidemiological studies have indicated a clear relationship between the development of allergic diseases and exposure to EDCs [111,112]. Evaluation of the effect of EDC exposure on infants was performed by comparing 73 bottle-fed and 98 breastfed children, correlating disease history within the early postnatal stage and the organochlorine concentration in milk. The result showed that prenatal exposure to dichlorodiphenyldichloroethylene and hexachlorobenzene was related to the risk of otitis media [113]. A study conducted to investigate the effect of gestational exposure to PCBs reported that the antibody response to diphtheria toxoid in 119 children exposed to PCBs during pregnancy decreased by 24.4% at 18 months of age. The same study confirmed that perinatal exposure to PCBs resulted in a 16.5% decrease in tetanus toxoid antibody response at 7 years of age in 129 children examined during the study [114]. A study conducted to assess the effect of BPA and triclosan on immune parameters in the US population indicated that the urinary concentration of BPA was correlated with elevated cytomegalovirus antibody titers, while the urinary concentration of triclosan showed a positive correlation with allergy and hay fever diagnoses. The author concluded that BPA and triclosan suppressed human immune function [115]. Lipophilic EDCs have also been reported to cause immunological disorders in infants [116]. An inverse association was reported between organochlorine pesticides and T helper cell type 1 in 31 randomly recruited women from Western Australia [117]. The suppression of T helper cells and skewed balance in Th1/Th2 are mechanisms by which EDCs compromise immune function [35]. Another study involving the analysis of whole blood samples from 349 children exposed to PCB indicated that postnatal exposure to PCB resulted in a fluctuation in lymphocyte subsets, suggesting the impairment of the postnatal immune system [118]. It is also worth noting that the complete lockdown implemented in most countries to contain the spread of the virus has forced many people to stock their houses with canned food, junk food, and food items preserved with potential EDCs. Consequently, many people may develop immunosuppression and be prone to severe SARS-CoV-2 infection owing to long-term exposure to EDCs.

## 4. Underlying Comorbidities of COVID-19

The principal causes of severe illness and fatality of COVID-19 are compromised host immunity and underlying comorbidities (a summary of selected clinical studies on COVID-19 comorbidities is shown in Table 1) [119,120,121,122,123,124,125,126,127,128]. Commonly reported underlying conditions in cases of severe COVID-19 and related death include diabetes, hypertension, cancer, immunodeficiency, cardiovascular diseases, obesity, and renal/kidney disease [120]. It has been shown that the clinical and treatment responses of COVID-19 patients with underlying disease as comorbidity were poorer than those of patients without comorbidities [124]. The authors further established a positive correlation between comorbidities and poor clinical outcomes. These diseases can also compromise the normal function of the immune system, creating a conducive environment for the invasion and progression of SARS-CoV-2 infection [129]. Besides, EDCs such as arsenic and dioxins have been shown to directly interfere with the host immune system, leading to its dysfunction [3,4,130,131,132,133].

## 5. Lesson from the Previous Pandemic

Infectious disease outbreaks have made significant impacts on societies in human history. Major recent pandemics include Asian flu (H3N2), severe acute respiratory syndrome (SARS), influenza (H1N1), middle east respiratory syndrome (MERS), Spanish flu, and severe acute respiratory syndrome coronavirus 2 (SARS-CoV 2). The contribution of environmental chemicals to the severity and comorbid diseases of these pandemics has been an oversight to researchers until recent years. Respiratory infections including H1N1, which spread worldwide in 2009, are notable public health concerns. It has been predicted that approximately 5–15% of the world population will contract influenza infection annually, leading to >3–5 million hospitalizations and 250,000–500,000 deaths worldwide [134]. Similar to COVID-19, H1N1 comorbid diseases included diabetes, chronic liver disease, kidney disease, heart disease, and cerebrovascular disease [135,136]. Meanwhile, unlike H1N1, a strong inflammatory response has been observed in COVID-19. In a study conducted to investigate the involvement of arsenic in the severity of H1N1 [3], C57BL/6J mice were exposed to 100 ppb arsenic for 5 weeks, followed by intranasal exposure to influenza A/PuertoRico/8/34 (H1N1) virus. Arsenic exposed mice infected with influenza A (H1N1) showed significant severe morbidity accompanied by ≥20% body weight loss and compromised immune response 8 days post-infection compared with their counterparts exposed to either arsenic or influenza type A virus only. The whole-lung homogenates levels of influenza A virus in arsenic exposed mice showed 10 times increase in viral titers correlating with their relative increase in morbidity [3]. In addition, the impairment of capillary functions, low cellular responses, reduced cytokine production, pulmonary edema, and hemorrhaging were observed in arsenic exposed mice [3]. Similarly, a dose-dependent rise in mortality occurred when female C57BL/6 mice were administered 1, 5, or 10 μg/kg body weight of TCDD a day before intranasal exposure to influenza A virus strain (A/HKx31) [131]. Moreover, a decrease in major ways of viral elimination (T-cell expansion, interleukin-2 (IL-2) and interferon gamma (IFNγ) production, and cytotoxic T lymphocytes) was observed in TCDD exposed mice [4]. Several other studies also confirmed that the exposure of mice to TCDD impairs many aspects of the host immune response to different strains of influenza A virus infections, resulting in suppressed virus-specific Immunoglobulin G (IgG) levels, enhanced pulmonary inflammation, and altered cytokine production in the lung and lymph nodes [130,131,132,133]. These reports may throw light on how EDCs influence clinical outcomes of infectious diseases in humans.

## 6. Current Knowledge on EDCs and COVID-19 Risks

Since COVID-19’s emergence in late 2019, EDCs have been speculated to be contributors to its risks [18]. The role of long-term exposure to toxic chemicals in COVID-19 clinical outcomes was reported to be grossly neglected, leading to the one sided biological approach of containment while the toxicological approach is abandoned [19]. Additionally, the spread and mortality rate of COVID-19 were presented as an opportunity to reassess the correlation between exposure to anthropogenic pollutants and pandemics [17]. Recently, a computational systems biology approach was used to study the relationship between EDCs and COVID-19 severity and identified the T-helper cell 17 (Th17) and the advanced glycation end products/receptor for advanced glycation end products (AGE/RAGE) pathways as principal targets through which EDCs could contribute to COVID-19 severity [20]. A non-mechanistic study that analyzed the urine and serum concentrations of Per- and poly-fluoroalkyl substances (PFASs) found a positive association between urinary levels of perfluorooctanesulfonic acid (PFOS) (odds ratio: 2.29 (95% CI: 1.52–3.22)), perfluorooctanoic acid (PFOA) (2.91, (1.95–4.83)), and total PFASs (Σ (12) PFASs) (3.31, (2.05–4.65)) with the risk of COVID-19 infection [137]. These preliminary studies provide insight into how EDCs can influence the clinical outcome of COVID-19 disease. However, the relationship between EDCs and COVID-19 risks still requires a comprehensive investigation.

## 7. Recommended Methodologies for Assessing the Role of EDCs in COVID-19 Severity

To comprehensively study the association between EDCs and COVID-19 severity, we recommend biomonitoring studies in different regions of the world in which the serum and urine levels of common EDCs of severely ill (intensive care) and asymptomatic or mild COVID-19 patients will be compared for differences. The study could be conducted before the end of the COVID-19 pandemic to obtain enough samples. The sample size in all regions should be large enough (for example, 200 or more patients per group) to avoid bias. Similarly, the serum and urine concentrations of EDCs in COVID-19 patients exhibiting poor and good response to treatments should be compared. Secondly, animal studies as adopted by Kozul et al. (2009) and Warren et al. (2000) [3,4] to investigate the involvement of arsenic and TCDD in the severity of H1N1 disease could be used to confirm the contribution of EDCs to COVID-19 severity. The study should be designed to expose laboratory animals to EDCs over a period and expose them to a mild strain of SARS-CoV 2. The severity of the infection and fatality will consequently be compared with their counterparts (control) that are not exposed to EDCs. Sources of bias such as age, sex, and strain should be minimized. Studies that adopt these designs will expand the current knowledge of EDCs with COVID-19 severity and will assist governments and policymaking agencies to enact necessary laws.

## 8. Conclusions

The leading underlying health conditions and comorbidities contributing to fatality due to many viral and bacterial infections, including COVID-19, are diabetes, obesity, cancer, cardiovascular disease, and immune dysfunction. These non-communicable diseases are on the rise in both developed and developing countries, and cannot be attributed only to genetics and nutrition. Daily exposure to EDCs via multiple sources contributes to the development of underlying health conditions, thereby increasing the severity of a pandemic, such as the COVID-19 pandemic. In this article, we have highlighted that the role played by EDCs in the development of comorbid disease and the impairment of the body’s immunity could have contributed to the severity and fatality of COVID-19. Although this review may not address the immediate crisis of the COVID-19 pandemic, it is important to know that other pandemics or epidemics may be encountered in the future, and a reduction in the level of daily exposure to EDCs will go a long way toward preventing fatalities. Similarly, non-communicable diseases that serve as underlying conditions during pandemic disease outbreaks have both environmental and genetic origins, and thus, reducing exposure to EDCs could greatly reduce the fatality of a future pandemic.

## Figures and Tables

**Table 1:** Selected clinical studies on major comorbidities of COVID-19 and clinical outcomes.

Sample Size (n)	Sex (%)	Sample Collection	Total Comorbidity (%)	Major Comorbidities (%)	Clinical Outcomes (%)	References
138	Male (54.3) Female (45.7)	Zhongnan Hospital, Wuhan, China	46.4	Hypertension (31.2) Cardiovascular disease (14.5) Diabetes (10.1) Malignancy (7.2) Cerebrovascular disease (5.1) Lung disease (2.9) Kidney disease (2.9) Liver disease (2.9)	ICU patients (26.08) Non-ICU patients (73.91)	Wang et al. [9]
41	Male (73.0) Female (27.0)	Jin Yin-tan Hospital, Wuhan, China	32	Diabetes (20) Hypertension (15) Cardiovascular disease (15) Lung disease (2) Liver disease (2) Malignancy (2)	ICU patients (31.70) Non-ICUpatients (68.29)	Huang et al. [11]
548	Male (50.9) Female (49.08)	Tongji Hospital, Wuhan, China	64.6	Hypertension (30.3) Diabetes (15.1) Cardiovascular disease (6.2) Lund disease (5.6) Carcinoma (4.7) Kidney disease (1.8) Liver disease (0.9)	Death (16.5) Severe cases (49.08) Non-sever cases (50.91)	Li et al. [12]
191	Male (62) Female (38)	Jinyintan Hospital and Wuhan Pulmonary Hospital, China	48	Hypertension (30) Diabetes (19) Cardiovascular disease (8) Lung disease (3) Carcinoma (1) Kidney disease (1)	Survivor (70.72) Non-survivor (28.27)	Zhou et al. [13]
663	Male (48.4) Female (51.6)	Renmin Hospital, Wuhan, China	37.3	Lung disease (7.7) Cardiovascular disease (24.7) Gastrointestinal disease (4.7) Endocrine disease (10.1) Kidney disease (3.2) Carcinoma (2.1) Inflammatory diseases (0.9)	Death (67.1) Mild cases (38.31) Severe cases (47.51)	Zhang et al. [14]
52	Male (67) Female (33)	Yin-tan hospital, Wuhan, China	40	Diabetes (17) Brain disease (13.5) Cardiovascular disease (10) Lung disease (8) Malignancy (4) Dementia (2) Malnutrition (2)	Died (61.53)	Yang et al. [119]
NM	NM	North Carolina, USA		Cardiovascular diseases (14.0) Diabetes (11.0) Lung disease (10.0) Kidney disease (3.0) Others (5.0)	NM	NCDHHS [120]
305	Male (49.5) Female (50.5)	A hospital in Georgia, USA	94.09	Diabetes (39.7) Cardiovascular disease (53.8) Lung disease (36) Obesity (12.7) Kidney disease (5.2) Liver disease (2.3)	Died (17.1) ICU patients (39) Non-ICU patients (61)	Gold et al. [121]
1099	Male (58.1) Female (41.9)	Wuhan Jinyintan Hospital, Wuhan, China	23.7	Hypertension (15.0) Diabetes (7.4) Cardiovascular disease (2.5) Liver disease (2.1) Lung disease (1.1) Carcinoma (0.9) Kidney disease (0.7)	Died (1.4) Non-severe cases (84.25) Severe cases (15.74)	Guan et al. [122]
74	Male (50.0) Female (50.0)	Hospitals in the Zhejiang Province, China	33.78	Hypertension (16.22) Diabetes (9.46) Liver disease (10.81)	Severe cases (22.97) Mild cases (77.97)	Jin et al. [123]
1590	Male (57.3) Female (42.7)	Throughout China	25.09	Diabetes (8.2) Hypertension (16.9) Chronic obstructive pulmonary disease (COPD) (NM) Carcinoma (NM)	Death (3.1) ICU patients (6.2)	Guan et al. [124]
99	Male (68) Female (32)	Jinyintan Hospital, Wuhan, China	51%	Cardiovascular disease (40) Digestive system disorder (11) Endocrine disorders (13) Carcinoma (1) Lung disease (1) Nervous system disease (1)	Died (11) Severe cases (58) Non-severe cases (31)	Chen et al. [125]
44,672	NM	Hubei Province, China	NM	Cardiovascular disease (10.5) Diabetes (7.3) Lung disease (6.3) Hypertension (6.0) Carcinoma (5.6)	Died (2.3) Mild cases (81.0) Severe cases (14.0) Critical cases (5.0)	Wu and McGooga [126]
140	Female (49.3) Male (50.7)	Hospitals in Wuhan, China	64.3	Hypertension (30.0) Diabetes (12.1) Liver disease (5.7) Gastrointestinal disease (5.0) Cardiovascular disease (8.5) Hyperlipidemia 7 (5.0) Endocrine diseases (3.6) Liver disease (1.4) Lung disease (1.4)	Non-severe cases (58.57) Severe cases (41.42)	
7162	NM	USA	37.6	Diabetes (10.9) Lung disease (9.2) Cardiovascular disease (9.0) Autoimmune disease (3.7) Kidney disease (3.0) Liver disease (0.6)	ICU patient (14) Non-ICU patient (28.55)	CDC [127]
202	Male (57.4) Female (42.6)	Jiangsu province, China	27.2	Hypertension (14.4) Diabetes (9.4) Lung disease (3.5) Liver diseases (2.0) Cardiovascular diseases (2.5) Cerebrovascular diseases (1.5) Carcinoma (1.0)	Death (0) Non-severe cases (88.61) Severe cases (11.38)	Huang et al. [128]

NM: Not mentioned. ICU: Intensive Care Unit. The percentage of dead patients is calculated within the selective period of time when the analysis was carried out; therefore, it may not represent the number of actual fatalities (%).

## Data Availability

Not applicable.
